# Validation of the Italian version of the "Mood Disorder Questionnaire" for the screening of bipolar disorders

**DOI:** 10.1186/1745-0179-1-8

**Published:** 2005-07-21

**Authors:** Maria Carolina Hardoy, Mariangela Cadeddu, Andrea Murru, Bernardo Dell'Osso, Bernardo Carpiniello, Pier Luigi Morosini, Joseph R Calabrese, Mauro Giovanni Carta

**Affiliations:** 1 Division of Psychiatry, Department of Public Health, University of Cagliari, Italy; 2 Department of Psychiatry, Neurobiology, Pharmacology, Biotechnology, University of Pisa, Italy; 3 National Institute of Health, Rome, Italy; 4 Case University School of Medicine Director, Mood Disorders Program, University Hospitals of Cleveland, USA

**Keywords:** bipolar disorder, mood disorders, screening, mood disorder questionnaire

## Abstract

The study measured the accuracy of the Italian version of the Mood Disorder Questionnaire (MDQ) as a screening instrument for bipolar disorders in a psychiatric setting.

**Methods:**

154 consecutive subjects attending the Division of Psychiatry of the University of Cagliari (Italy), were screened for bipolar disorders using the Italian translation of the MDQ, and diagnostically interviewed with the Structured Clinical Interview for DSM-IV Axis I Disorders (SCID) by physicians.

**Results:**

On the basis of the SCID: 51 (33.1%) received a diagnosis of bipolar or schizoaffective bipolar type disorders, 63 (40.9%) were diagnosed as having at least one psychiatric disorder in Axis I (other than bipolar or schizoaffective bipolar type disorders), whilst 40 (25.9%) were unaffected by any type of psychiatric disorder. MDQ showed a good accuracy for bipolar or schizoaffective bipolar type disorders: the cut-off 4 had sensitivity 0.90 and specificity 0.58; the cut-off 5 had sensitivity 0.84 and specificity 0.70; and the cut-off 6 had sensitivity 0.76 and specificity 0.86. The accuracy for bipolar II disorders was sufficient but not excellent: the cut-off 4 had sensitivity 0.80 and specificity 0.45; the cut-off 5 had sensitivity 0.70 and specificity 0.55; and the cut-off 6 had sensitivity 0.55 and specificity 0.65.

**Conclusion:**

Our results seem to indicate a good accuracy of MDQ, and confirm the results of recent surveys conducted in the USA. Moreover the instrument needs to be validated in other settings (e.g. in general practice).

## Background

Bipolar disorders are recurring psychiatric conditions, often of a chronic nature and highly invalidating. This type of disorder is not always correctly diagnosed, thus delaying the administration of an efficient means of treatment [1].

The difficulties encountered in correctly recognising bipolar disorders are possibly greater in epidemiological studies or for screening before psychiatric evaluation in medical settings where clinical interviews tend to be substituted by the use of a standardised rating tool; in this instance the interviewer (not a specialised psychiatrist) is unable to identify aspects which are fundamental for a correct diagnosis of the illness.

On the basis of the above considerations, and in view also of the high cost of involving specialised psychiatrists in studies of an epidemiological nature (at times carrying out a semi-structured interview), international research has focused on the compiling of screening questionnaires to be used in epidemiological investigations of bipolar disorders, to be performed according to a "2nd phase" design.

The aim of the present study, carried out in a sample of psychiatric patients attending a mental health clinic with an elevated proportion of patients coming from the general hospital for a psychiatric evaluation, was to obtain a preliminary standardisation of the Italian version of one of the most recently prepared screening tools, the Mood Disorder Questionnaire (MDQ) [2-4]. The "Gold Standard" is represented by a psychiatric diagnosis carried out by means of a semi-structured interview.

## Methods

### Study design

The study design consisted in the evaluation of the accuracy of the Italian version of the Mood Disorder Questionnaire (MDQ) [2-4], using the Structured Clinical Interview for DSM-IV Axis I Disorders (SCID) [5] as a Gold Standard. The SCID was held with all subjects physicians working in the field of psychiatry for at least three years, all of whom had undergone specific training for the use of the SCID.

Immediately prior to the interview all subjects had filled in the Italian version of the MDQ. Written informed consent was obtained from all study participants. The questionnaire had been translated into Italian before the start of the research project, had been back-translated into English and approval had been obtained from one of the authors of the original version.

Table 1 illustrates the characteristics of the sample: a consecutive series of 154 subjects (61 males, mean age 35.9 ± 12.3 years; 93 females, mean age 38.4 ± 12.5 years) referred to the Division of Psychiatry of the University of Cagliari (Italy) either: seeking psychiatric care or counselling, coming from the general hospital of the University of Cagliari for a psychiatric evaluation, or applying for legal certification of their mental capacities (for driving and/or gun licences, etc).

**Table 1:** Characteristics of the sample.

Sample	N	Mean age ± SD (years)
Males	61	35.9 ± 12.3
Females	93	38.4 ± 12.5
Total	154	37.2 ± 12.4

### Instruments

SCID: the Structured Clinical Interview for DSM-IV Disorders of Axis I [5] is a semi-structured interview aimed at formulating the main diagnoses covered by Axis I of DSM-IV [6].

MDQ: the Mood Disorder Questionnaire [2-4] is a self-administered single-page paper and pencil inventory made up of 13 yes/no items derived from both the DSM-IV criteria [6] and clinical experience, which assess the macro-area of mood, a lifetime history of a manic or hypomanic syndrome, placing particular emphasis on a marked subjective variation in the dimensions of irritability, activity, sociability, sleep, libido, thoughts, attention, energy, behaviour, etc.

We evaluated the discriminatory capacity (patients with a diagnosis of Bipolar Disorder I or II or Schizoaffective Disorder Bipolar type versus patients with other psychiatric diagnoses or with no psychiatric diagnosis, according to the findings of the SCID) of all 13 items contemplated in the MDQ.

The accuracy of the MDQ questionnaire was calculated in terms of sensitivity and specificity for each theoretically possible cut-off point (number of positive answers). Overall performance of the questionnaire was graphically assessed by means of the Relative Operating Characteristic Analysis [7]. The specific accuracy for detecting bipolar II disorders was calculated at best performing cut-off points 4, 5 and 6.

## Results

**Figure 1:**
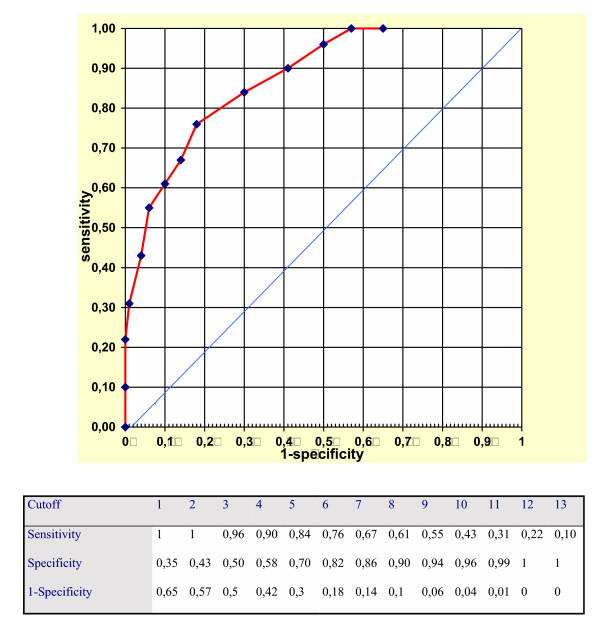
Table 3

Table 2 shows the psychiatric diagnoses in Axis I formulated on the basis of the SCID; out of all subjects interviewed (total N = 154): 51 (33.1%) received a diagnosis of bipolar or schizoaffective bipolar type disorders, 5 of these (9.8%) were schizoaffective bipolar type, 26 (51.0%) were bipolar I and 20 (39.2 %) were bipolar II; 63 (40.9%) were diagnosed as having at least one psychiatric disorder in Axis I (other than bipolar or schizoaffective bipolar type), whilst 40 (25.9%) were unaffected by any type of psychiatric disorder. Table 3 (See Figure 1)illustrates the performance of the MDQ by means of ROC analysis. The accuracy for bipolar II disorders was: at the cut-off 4 sensitivity 0.80 and specificity 0.45; at the cut-off 5 sensitivity 0.70 and specificity 0.55; and at the cut-off 6 sensitivity 0.55 and specificity 0.65.

**Table 2:** Psychiatric diagnoses in Axis I formulated according to findings of the SCID semi-structured interview [5].

PSYCHIATRIC DIAGNOSES IN AXIS I						
Bipolar or Schizoaffective disorder		At least another 1 disorder in Axis I		No diagnosis		Total subjects

N	%	N	%	N	%	N
51	33.1	63	40.9	40	25.9	154

**Table 3:** Performance of the MDQ by means of ROC analysis.

Cutoff	1	2	3	4	5	6	7	8	9	10	11	12	13
Sensitivity	1	1	0,96	0,90	0,84	0,76	0,67	0,61	0,55	0,43	0,31	0,22	0,10
Specificity	0,35	0,43	0,50	0,58	0,70	0,82	0,86	0,90	0,94	0,96	0,99	1	1
1-Specificity	0,65	0,57	0,5	0,42	0,3	0,18	0,14	0,1	0,06	0,04	0,01	0	0

Please see Figure 1.

## Discussion

The results of this study seem to indicate a fairly good performance of the questionnaire, at least from a preliminary point of view. The use of different cut-off points depends on use intended for a questionnaire. For two phases investigations, in which it is of the utmost importance that "cases" not be overlooked during screening, as all positive cases will subsequently undergo clinical assessment, it is necessary to reduce the "false negatives" to a bare minimum. It is therefore preferable to use a very high sensitivity, even though this may prejudice the specificity. Cut-off point 4 of MDQ (sensitivity = 0.90, specificity = 0.58) would for example allow an accurate two-stage investigation to be carried out, whilst reducing interviews by more than 50%. This cut-off seems to be recommended for a screening in a psychiatric setting with a high number of psychiatric evaluations from the general hospital as the psychiatric unit where this study was performed. At this cut-off for detecting bipolar II disorders is sufficient particularly concerning the good sensitivity of 0.80 and thus the low rate of false negatives. Nevertheless the accuracy concerning bipolar II disorders is not excellent and is necessary to underline that in tertiary care, bipolar II patients are more likely to have a cyclothymic temperament (i.e. a baseline high instability), which makes more likely to have some impairment during hypomania [8]. But these patients are a minority compared to the community and non-tertiary care patients. Bipolar II often showing improved functioning (in non-tertiary care outpatients) was recently reported by Benazzi [9]. MDQ may underdiagnose bipolar II disorder because it requires moderate/severe impairment to score positive. Thus the validation of MDQ in the psychiatric setting does not justify the use of this tool in other settings (i.e. in general practice or in the community).

The MDQ has been used in the United States as part of a nationwide telephone investigation of the general population, according to a single phase survey. This research study was aimed at identifying "cases" treated and quantifying the impact and social cost of bipolar disorders. For a one-stage study to be carried out in the general psychiatry setting the ideal cut-off point could be 6 (sensitivity 0.76, specificity 0.82). The latter is the nearest point to the 0.1 vertex of the ROC diagram. Indeed, this point represents the ideal screener (sensitivity 1, specificity 1). On the other hand, the area depicted by the curve beyond the diagonal of the diagram represents the area of validity of the screener. At this cut-off the accuracy for bipolar II disorders remains sufficient (sensitivity 0.55 and specificity 0.65) for research studies but not for screening due to the low sensitivity and, consequently, the high rate of false negative.

It should moreover be underlined that the MDQ is a simple and easy-to-use tool. Our results seem to indicate a good applicability of MDQ in the European clinical settings, and confirm the recent survey carried out in Finland [10].

## Competing interests

The author(s) declare that they have no competing interests.

## Authors' contributions

MCH, MGC, PLM conceived of the study, participated in the design of the study, coordinated the study, performed the statistical analysis and drafted the manuscript. MC, AM, BDO, BC, JRC participated in its design and coordination. All authors read and approved the final manuscript.
